# Analysis of injury death trends among women in Macheng City, China, 1984-2008

**DOI:** 10.1186/1471-2458-11-698

**Published:** 2011-09-13

**Authors:** Yang Hu, Li Wu, Xiang Yu, Dekai Zhang, Xiaoxian Liu, Youjie Wang

**Affiliations:** 1 Department of Maternal and Child health, School of Public Health, Tongji Medical College, Huazhong University of Science and Technology, Hangkong Road 13, Wuhan, 430030, China; 2 Maternal and Child Hospital of Jiangxi Province, Bayi Avenue 318, Nanchang, 330006, China; 3 Tongji Center of Injury Prevention, Tongji Medical College, Huazhong University of Science and Technology, Hangkong Road 13, Wuhan, 430030, China; 4 Health Department of Macheng city, Jiangjun North Street 117, Macheng, Hubei Province, 438300, China

**Keywords:** women's injury, death trend, suicide

## Abstract

**Background:**

There are few studies on trends in injury death rates in China during the recent decades of tremendous change in this society. This paper examined trends in injury mortality rates among women aged 15 years or older in Macheng City from 1984-2008.

**Methods:**

Data on injury deaths in women from 1984 to 2008 were obtained from the Death Registry System in Macheng City. Injuries were classified using the International Classification of Diseases (ICD), 9th and 10th editions.

**Results:**

The average overall injury death rate of women aged 15 or older was 87.6/100,000 in Macheng City from 1984-2008. Injuries were the leading cause of death for women aged 15-44 and the fourth cause of death for all women during this period. The all-cause injury mortality rate decreased from 149.01 per 100,000 population in 1984 to 32.90 per 100,000 population in 2008 for women. Road traffic injury (RTI) was the only injury for which the mortality rate increased dramatically from 1984 (1.35 per 100,000) to 2008 (4.63 per 100,000). For all age groups, suicide was the leading cause of injury death. For women aged 15 to 64, RTI and drowning were the second and third leading causes of injury deaths, respectively.

**Conclusions:**

The injury mortality rate for women aged 15 years or older decreased by 77.92% from 1984-2008. In contrast, RTI deaths increased sharply in the 2000s compared with the 1980s. Although the suicide rate decreased dramatically, it was still the leading cause of injury death for women. Research is needed to identify risk factors contributing to the increase in RTI and decrease in suicides.

## Background

Injuries have been shown to account for a significant health burden on all populations, regardless of age, sex, income, or geographical region [1]. Around the world, almost 16,000 people die from injuries every day. In China, injuries have emerged as a significant public health problem. It has been estimated that each year injuries claim about 700,000 lives, account for 9% of all-cause deaths, and is the fifth leading cause of death among people in China [2,3]. The Chinese society has undergone many changes during recent decades. Little is known regarding the impact of rapid economic growth, urbanization, housing, and education as well as an increased number of roads and motor vehicles on injury profiles in China.

Studies both in China and other countries have shown that there are differences in mortality and patterns of injury in men and women [4,5]. Although men are more likely than women to die of injury, injury in women is also a severe worldwide problem. Injuries are the leading cause of death for females 1-34 years of age and are responsible for more years of potential life lost than any other cause of death. Discerning injury profiles and trends are essential for the adoption of adequate policy and planning for injury prevention and control.

Women in Macheng City have an extremely high suicide death rate compared to women in other parts of China [6,7], and have a similar injury death rate as their male counterparts. We investigated distinct patterns of injury death in women in Macheng City, using the city's reliable death registry system. Examining injury trends has important policy implications for injury prevention. In this study, we used data from the death registry system to examine injury trends and characteristics of injury in women aged 15 years or older in Macheng City from 1984-2008.

## Methods

### Data Source

Injury death data on residents of Macheng City were obtained from the death registry system. The death database included information on primary cause of death, death date, sex, and age. The mortality data from the death registry system were categorized based on the International Classification of Diseases 9th (ICD-9) and 10th (ICD-10) editions, depending on the year in which the data were released. In 2003, the system for coding deaths in Macheng City changed from ICD-9 to ICD-10. The ICD-9 and ICD-10 codes were grouped in this study into road traffic injury (RTI; E800-E848 for ICD-9; V01-V89, V99, Y850 for ICD-10), poisoning (E850-E869 for ICD-9; X20-29, X40-49 for ICD-10), fire/flame injury (E890-E899 for ICD-9; X00-X09 for ICD-10), drowning (E910 for ICD-9; W65-W74 for ICD-10), suicide (E950-E959 for ICD-9; X60-X84, Y870 for ICD-10), and falls (E880-E888 for ICD-9; W00-W19 for ICD-10). Other causes of injuries and ill-defined cause injuries were defined as "others". Injuries grouped into "others" accounted for 5.35% of all injuries in this study. In this death registry system, people died with an intention to kill him/herself using the method of traffic accident, poisoning (i.e pesticide), fire/flame, drowning, or fall was coded as suicide.

The annual midyear population figures in 1984-2008 were obtained from the Census Bureau of Macheng City to calculate injury mortality rates per 100,000 women aged 15 or older.

Macheng City is located in northeastern Hubei province. The population was 1.21 million, with 70% of the people living in rural areas in 2008. This city established the earliest death registry system in China in 1974 under the guidance and assistance of the faculty from Tongji Medical College. Statisticians from the Department of Epidemiology and Biostatistics of Tongji Medical College have trained the staff working for the system, supervised the process of data collection, and verified the data by sampling each year since 1974. To the best of our knowledge, the death registry system in Macheng city is the only one not only supervised by provincial professionals, but also by statistical professionals from a medical college in China. To date, more than 20 papers have been published using data from this death registry system. The data from this system were considered complete and reliable for this study. The most recent continuous period for which complete individual records of death were computerized was 1984-2008.

### Statistical Analysis

The injury mortality rate for women 15 years of age or older are expressed as the number of cases per 100,000 persons. Age-adjusted rates were calculated by a direct method standardized to the total Chinese population in 1990.

Ethics approval for of this research was obtained from the Research Ethics Committee of Tongji Medical College, Huazhong University of Science and Technology.

## Results

From 1984 to 2008, there were a total of 10,915 injury deaths in women aged 15 years or older in Macheng City. The overall injury death rate of women was 87.60/100,000. As shown in Table 1, injury was the fourth leading cause of death overall during 1984-2008 among women older than age 15. Injury was the leading death cause for women aged 15-44. Injury mortality increased sharply after the age of 55. It was highest in the 75 and older age group and lowest in the 15-24 age group.

**Table 1:** Ranking of injury in all-cause death, by age group, 1984-2008

Age	All-cause mortality	Injury mortality rate	Injury death as of %	Rank
	(1/100 000, 95%CI)	(1/100 000, 95%CI)	all-cause death	
15-	135.18(130.64~139.71)	93.59(89.82~97.37)	69.33	1
25-	163.49(158.25~168.72)	92.73(88.78~96.67)	56.89	1
35-	254.78(247.29~262.27)	81.92(77.67~86.18)	32.13	1
45-	618.48(604.77~632.19)	94.51(89.13~99.88)	15.29	4
55-	1788.49(1760.84~1816.15)	161.05(152.68~169.42)	9.01	5
65-	4428.81(4373.67~4483.96)	269.70(255.78~283.62)	6.09	5
75-	10884.38(10741.74~11027.02)	497.43(465.13~529.73)	4.57	4
Total	837.14(831.32~842.96)	115.54(113.37~117.71)	13.8	4

**Figure 1:**
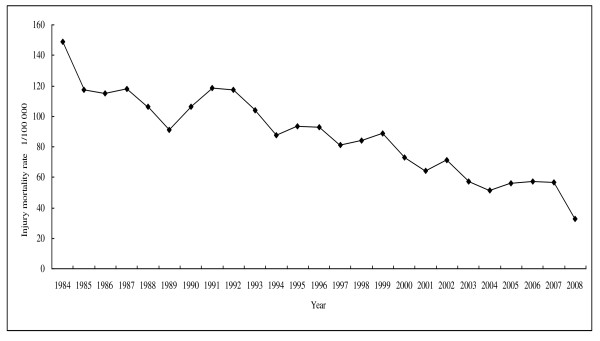
**The trends of all cause injury mortality rate (1/100 000) in Macheng city, 1984-2008**.

Over the study period, there was a dramatic decline in annual number of deaths and age standardized mortality rates for all-cause injury. Our study showed the trend in age-adjusted injury mortality rate per 100,000 population for women older than age 15 (Figure 1). The mortality rate decreased from 149.01 per 100,000 in 1984 to 32.90 per 100,000 in 2008, reflecting a reduction rate of 77.92%.

**Figure 2:**
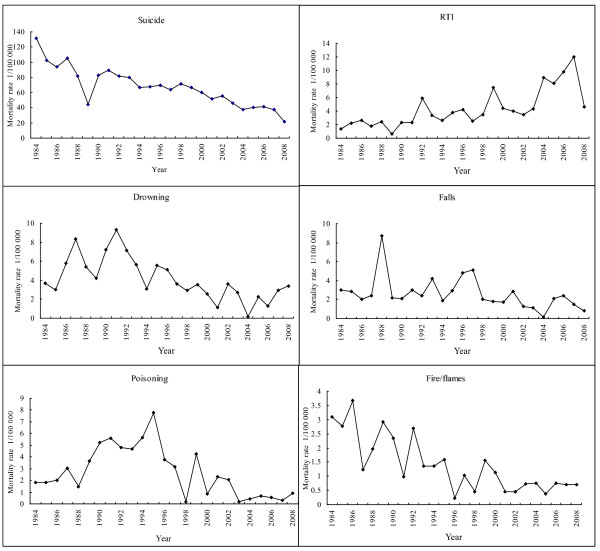
**The trends of cause-specific injury mortality rates (1/100,000) in Macheng city, 1984-2008**.

We analyzed cause-specific injury mortality rates for women from 1984 to 2008 (Figure 2). During the study period, the mortality rates for most cause-specific injuries showed a decreasing trend, while the mortality rate for RTI increased from 1.35 per 100,000 in 1984 to 4.63 per 100,000 in 2008. As seen in Figure 2, we also found that the mortality rate from suicide was consistently higher than from other causes of injury. In the mid 1990s, RTI surpassed drowning and became the second leading cause of injury in women.

As shown in Table 2, suicide was the leading cause of injury in women for each age group. We found that women older than age 75 had the highest suicide rate. Among women aged 15-64, RTI and drowning were the second and third leading causes of injury deaths, respectively. Among women aged 65-74, the second and third injury death causes were drowning and falls, respectively. For women older than 75, falls and fire/flames were the second and third causes of injury death, respectively.

**Table 2:** Ranking of leading cause of injury death, by age group, 1984-2008 (1/100000, 95%CI)

Age	1	2	3	4	5
15-	suicide	drowning	RTI	poisoning	fall
	73.92 (70.56~77.27)	5.99(5.81~8.31)	3.93 (3.15~4.70)	2.86 (2.20~3.52)	1.03 (0.64~1.43)
25-	suicide	RTI	drowning	poisoning	fall
	74.38 (70.85~77.92)	4.98 (4.07~5.89)	3.80 (3.00~4.60)	3.36 (2.61~4.11)	1.62 (1.10~2.14)
35-	suicide	RTI	drowning	poisoning	fall
	61.64 (57.95~65.33)	7.07 (5.82~8.31)	3.27, 2.42~4.12	2.24 (1.54~2.94)	1.67 (1.06~2.27)
45-	suicide	RTI	drowning	fall	poisoning
	69.43 (64.82~74.04)	8.04 (6.47~9.61)	3.58 (2.54~4.63)	2.87 (1.93~3.80)	2.79 (1.86~3.71)
55-	suicide	RTI	falls	drowning	poisoning
	125.46 (118.07~132.85)	7.82 (5.97~9.67)	6.12 (4.49~7.75)	5.55,4.00~7.12	5.10 (3.61~6.59)
65-	suicide	drowning	fall	fire/flames injury	poisoning
	201.80 (189.76~213.84)	13.47 (10.36~16.58)	11.22 (8.38~14.06)	10.47 (7.73~13.22)	8.04 (5.64~10.45)
75-	suicide	fall	fire/flames injury	drowning	RTI
	291.03 (266.32~315.73)	28.94 (21.15~36.73)	27.30 (19.73~34.87)	25.12 (17.86~32.38)	18.02 (11.87~24.17)

## Discussion

Our findings revealed that from 1984 to 2008 the injury mortality rate decreased substantially among women aged 15 or older. The total injury mortality rate in Macheng City decreased from 149.01/100,000 in 1984 to 32.90/100,000 in 2008, with a reduction of 77.92% during those years.

The decline in injury death rate most likely resulted from improvement in economic and social status. With one of the fastest growing economies in the world, China has experienced tremendous socioeconomic changes since the economic reforms in 1978. Its rapid growth has been accompanied by substantial changes in mode of transport, housing, and other ways of life, all of which affect exposure to risk factors for injury [8]. There is reportedly an inverse association between economic development and unintentional injury mortality among children and adults in low and middle income countries [9]. The decrease in mortality rate can also be attributed to the dramatic decrease in suicide mortality. As found in our study, suicide accounted for 77.2% of all injury deaths and showed a dramatic decrease during the study period. The suicide rate decreased from 131.1/100,000 in 1984 to 21.9/100,000 in 2008. Our findings are consistent with Chinese national data. Wang et al. [8] reported that rates of injury-related deaths in China decreased steadily from 1987-2006. Although the injury mortality rate for women decreased sharply during our study period, it was still higher than the injury mortality rates of most developed countries and the national rate in China [10].

Suicide is a major cause of premature death and thus is an important public health issue worldwide, particularly in China [6]. In this study, we found suicide was the leading cause of injury death in all age groups of women older than age 15 during the whole study period. This finding is consistent with data from most studies in China [11,12]. However, the suicide rate for women in Macheng City was higher than most suicide rates obtained in other studies conducted in China [8,13,14]. Phillips et al [6] reported an average suicide mortality rate for females of 25.9 per 100,000 yearly during the period of 1995-1999 in China. This rate was higher than that in other countries in the world [10]. In most countries, mortality rates due to suicide are higher among men than women. However, this relationship is reversed in China. Suicide fatalities are more common among women than men, and the highest suicide rate is found in rural women [6,8,13,15]. In Macheng City, the suicide rate for females was 1.3 times higher than of male counterparts (64.6/100,000 vs. 49.5/100,000) during 1984-2008. This unique pattern of suicide in China is attracting attention and requires further investigation. It is speculated that the widely available and common use of pesticides and insufficient emergency medical services in rural areas account most for the high mortality rate of suicide in China [16,17]. One study in Macheng City reported that 80.4% of suicide completers died by ingesting pesticides in 1992-1994 [18]. Another study reported that 77.9% of suicides in women in Macheng City were attributable to pesticide self-poisoning [19]. In addition, interpersonal conflict and disease as well as social and environmental factors such as economic hardship may be contributing factors [20]. Although suicide was the most common cause of injury death for women, our study revealed a significant improvement in the suicide mortality rate over time. We speculate that the decrease in suicides might be due to social changes, such as general improvement in living conditions, better education of women, small family size, more job opportunities for women, divorce made easier, and improvement of emergency medical services in cases of self-poisoning. To formulate effective strategies for suicide prevention, further research is required to delineate the mechanisms underlying the downward trend in suicide rate.

The most dramatic change in injury mortality during 1984-2008 was in RTI rate, which increased in most groups of women. In 1984, the mortality rate for RTI in Macheng City was lower than that in the USA (1.35 vs. 12.12 per 10,000 population). In 2007, it was higher than that in the USA (12.48 vs. 9.98 per 10,000 population) [21]. The alarming rise in RTI deaths is probably the consequence of the rapid increase in motor vehicles. The number of civil vehicles increased from 13,556 in 1985 to 1,368,635 in 2008 in Hubei province [22,23]. The rise in RTI deaths may also be associated with poor road conditions, less police supervision on the roads, insufficient emergency medical services, and higher rates of driving under the influence of alcohol. As the mortality rates for other causes of injury decreased during the study period, the RTI mortality rate increased sharply. However, we found that the mortality rate for RTI sharply decreased in 2008. The decreased RTI rate might be explained by the Road Safety Campaign initiated in 2008 in Macheng City to curb the trend of increased RTI mortality. The countermeasures included in this campaign were mandatory use of safety belts or helmets, and punishment for drunk driving and high speed driving.

We also observed that drowning was another major cause of injury death. Similar findings have been reported by Liu and Ni [24,25]. Contributing factors may be a combination of geographical features of Macheng City, including many ponds, lakes, and natural waterways. In addition, swimming is a popular activity in this city.

When we analyzed the ranking of leading causes of injury, we found that for women older than age 65, fire/flames and falls were the major causes besides suicide. Falls are one of the most common causes of injury in older people, especially for older women [26-28]. It has been reported that the fall-related injury rate in women older than age 65 is significantly higher than in men of comparable age [29]. In our study, fire/flames was another common cause of injuries in women older than age 65. The reasons for high mortality due to fire/flames among women are unclear. However, older women spend more time at home. When they are threatened by fire, they may not have enough time to escape because of muscle weakness and loss of lower body strength.

There are several limitations in this study. First, two systems (ICD-9 for 1984-2002, ICD-10 for 2003-2008) were used for disease coding during the study period. A potential problem occurs if a single injury is coded differently under the two systems. Although the disease coding staff were trained and certified before using the ICD-10, aligning the causes of mortality in the ICD-9 to those in the ICD-10 proved to be a complex issue and presented many challenges. Even though there was no unusual change in rate between 2002 and 2003 in this paper, we could not rule out the possibility of miscoding of injuries due to the coding system transition. The second limitation is that our study was based on data from the death registry system, which does not contain relevant information such as socio-economic status and lifestyle risk factors that would allow us to further analyze causes of injury. The third limitation is that the findings do not represent the entire Chinese population, and cannot be generalized to the overall Chinese population. However, considering the incompleteness of the death registry system in China, results of our study may be supplementary to most studies based on data from the Chinese Ministry of Health Vital Registration System or Disease Surveillance Point system.

Based on the findings of our research, targeted prevention strategies need to be adopted for reducing injury mortality. In the present study, suicide rates dramatically increased with age. Based on previous studies conducted in Macheng City [18,19], suicide risk among elderly women could be a consequence of increased severe illness and high medical cost. Providing medical insurance and financial support for elderly rural women might be an effective way to reduce the high suicide rate. Moreover, the divergent age patterns for different types of injury indicate that injury control programs should be age appropriate. For instance, drowning prevention should target middle-aged women, and fall prevention programs should focus on women older than age 65. Increasing mortality rates for RTI found during the study period suggest the need for more education programs and counseling for drivers on safe driving. Intervention strategies are urgently needed to address the high mortality rate from suicide injuries in women older than 15 years of age.

## Conclusions

We analyzed trends in injury death rates among women in Macheng City in 1984-2008 to increase awareness of the importance of injuries in women and used injury statistics for discussions about preventive action. During 1984-2008, injuries were the leading cause of death for women ages 15-49 and the fourth leading cause of death overall for women older than age 15. The age-standardized mortality rate due to injury for women older than age15 decreased substantially, but there was still a high prevalence of injuries. Suicide was the leading cause of injury for women older than age 15, and the mortality rate was higher than that for men. The cause-specific injury mortality rate decreased during 1984-2008 except for RTI in Macheng City. Results in this study provide a sound basis for measures that can be taken by government agencies to reduce the number of injury deaths among women.

## Competing interests

The authors declare that they have no competing interests.

## Authors' contributions

YW and YH, LW designed the study, performed the analysis, interpreted the data, and drafted the manuscript. XY analyzed the data and interpreted the results. DZ assisted with the design, conception, analysis, and interpretation of the data. XL reviewed and refined the manuscript. All authors read and approved the final manuscript.

## Pre-publication history

The pre-publication history for this paper can be accessed here:

http://www.biomedcentral.com/1471-2458/11/698/prepub
